# Sensitization-Associated Post-COVID-19 Symptoms at 6 Months Are Not Associated with Serological Biomarkers at Hospital Admission in COVID-19 Survivors: A Secondary Analysis of a Cohort Study

**DOI:** 10.3390/jcm11123512

**Published:** 2022-06-18

**Authors:** César Fernández-de-las-Peñas, Manuel Herrero-Montes, Diego Ferrer-Pargada, Sheila Izquierdo-Cuervo, Lars Arendt-Nielsen, Jo Nijs, Paula Parás-Bravo

**Affiliations:** 1 Department of Physical Therapy, Occupational Therapy, Physical Medicine and Rehabilitation, Universidad Rey Juan Carlos (URJC), 28922 Madrid, Spain; 2 CNAP, Center for Sensory-Motor Interaction (SMI), Department of Health Science and Technology, Faculty of Medicine, Aalborg University, DK-9220 Aalborg, Denmark; lan@hst.aau.dk; 3 Departamento de Enfermería, Universidad de Cantabria, 39008 Santander, Spain; manuel.herrero@unican.es (M.H.-M.); paula.paras@unican.es (P.P.-B.); 4 Grupo de Investigación en Enfermería, Instituto de Investigación Sanitaria Valdecilla (IDIVAL), 39011 Santander, Spain; 5 Servicio de Neumología, Hospital Universitario Marqués de Valdecilla, 39008 Santander, Spain; diegojose.ferrer@scsalud.es (D.F.-P.); sheila.izquierdo@scsalud.es (S.I.-C.); 6 Department of Medical Gastroenterology, Mech-Sense, Aalborg University Hospital, DK-9000 Aalborg, Denmark; 7 Pain in Motion Research Group (PAIN), Department of Physiotherapy, Human Physiology and Anatomy, Faculty of Physical Education & Physiotherapy, Vrije Universiteit Brussel, 1050 Brussels, Belgium; jo.nijs@vub.be; 8 Department of Physical Medicine and Physiotherapy, University Hospital Brussels, 1050 Brussels, Belgium; 9 Unit of Physiotherapy, Department of Health and Rehabilitation, Institute of Neuroscience and Physiology, Sahlgrenska Academy, University of Gothenburg, 40530 Gothenburg, Sweden

**Keywords:** COVID-19, sensitization, post-COVID-19, biomarkers, hospitalization

## Abstract

Individuals who survived coronavirus disease, 2019 (COVID-19), often have symptoms of sensitization, but the extent to which these symptoms relate to serological biomarkers remains unclear. Therefore, this secondary analysis evaluated the association between serological biomarkers at hospital admission with sensitization-associated post-COVID-19 symptoms in a sample of previously hospitalized COVID-19 survivors. Sixty-seven individuals hospitalized due to SARS-CoV-2 infection in one urban hospital of Madrid (Spain) during the first wave of the pandemic were assessed a mean of 6.0 (SD 0.8) months after hospital discharge. The Central Sensitization Inventory (CSI) was used as rough tool to estimate the presence of sensitization-associated post-COVID-19 symptoms (≥40/100 points). Levels of 16 serological biomarkers collected at hospital admission were obtained from medical records. Twenty-four (35.8%) patients reported sensitization-associated post-COVID-19 symptoms (CSI ≥ 40 points). Subjects reporting sensitization-associated symptoms had lower ferritin and hemoglobin levels than those not reporting sensitization-associated post-COVID-19 symptoms; however, these differences were small. We observed significant but small negative associations of the CSI score with ferritin (r: −0.251, *p* = 0.04) and hemoglobin (r: −0.292, *p* = 0.017) levels. No other significant difference was found. In conclusion, this secondary analysis did not find significant associations between the investigated serological biomarkers at hospital admission and sensitization-associated post-COVID-19 symptoms at 6 months after hospitalization in COVID-19 survivors.

## 1. Introduction

Recent data suggest that pain is a common post-COVID-19 symptom experienced by individuals who had survived severe acute respiratory syndrome coronavirus 2 (SARS-CoV-2) [1]. A cohort study proposed that post-COVID-19 pain primarily resembles musculoskeletal features [2]. Additionally, others have reported the presence of multiple and different pain symptoms [3], clinically mimicking the widespread pain features of fibromyalgia syndrome in up to 30% of COVID-19 survivors [4]. Musculoskeletal pain is usually associated with sensitization [5], which is an underlying characteristic of nociplastic pain [6]. Nociplastic pain, in addition to exaggerated pain responses, is also associated with central-nervous-system-derived symptoms, including fatigue, sleep problems, memory loss, and mood disturbances [7]. All these symptoms are also typically experienced by long haulers. Studies investigating the presence of sensitization in people with post-COVID-19 pain are scarce. Goudman et al. reported that 70% of post-COVID-19 patients showed sensitization-associated symptoms, based on a score of ≥ 40 points in the central sensitization inventory (CSI) [8]. A recent study reported that the prevalence of sensitization-associated symptoms (CSI score ≥40 points) was 33.7% [9]. Identification of those factors associated with the development of sensitization in COVID-19 survivors could help in identifying individuals at a higher risk, and, therefore, aid in implementing timely interventions. Given the close interplay between the immune system, pain, and sensitization [10,11], immune responses associated with SARS-CoV-2 may well generate immune factors (e.g., proinflammatory cytokines, elastase produced by activated leukocytes, oxidative stress) that potentially contribute in developing or sustaining sensitization, especially if they cross the blood–brain barrier. Hence, specific serological biomarkers at the acute phase of SARS-CoV-2 infection could be a potential risk factor contributing to the development of post-COVID-19 pain [12]. Here, we present a secondary analysis of a previous cohort study investigating the prevalence of sensitization-associated symptoms in previously hospitalized COVID-19 survivors exhibiting de novo post-COVID-19 pain [9]. The aim of this secondary analysis was to investigate the association between specific serological biomarkers at hospital admission with sensitization-associated post-COVID-19 pain symptoms. 

## 2. Methods

A secondary analysis of a previous observational cross-sectional cohort study was conducted [9]. Briefly, patients hospitalized during the first wave of the pandemic at an urban hospital in Spain due to SARS-CoV-2 infection, attending a specific post-COVID-19 unit from 1 June to 31 October 2021, were invited to participate. They were included if they reported pain as their primary post-COVID-19 symptom and did not present a preexisting history of pain symptoms or any medical comorbidity explaining the presence of pain, as previously described [9]. The Institutional Ethics Committee of INDIVAL Cantabria (code 2020.416) approved the study. All participants provided their informed consent. As previously described in detail [9], participants completed the following self-reported questionnaires: the CSI for evaluating sensitization-associated symptoms [13,14]; the 11-item Tampa Scale for Kinesiophobia for assessing the presence of fear of movement [15]; the Pain Catastrophizing Scale [16]; the Hospital Anxiety and Depression Scale for assessing the presence of anxiety or depressive levels [17]; and the paper-based 5-level version of EuroQol-5D (EQ-5D-5L) [18]. In this secondary analysis, we used a cutoff score of >40 points on the total score of the CSA (range: 0–100) for determining the presence of sensitization-associated symptoms [19]. We obtained the following serological biomarkers collected at hospital admission from hospital medical records: glucose, creatinine, aspartate transaminase (AST), alanine transaminase (ALT), lactate dehydrogenase (LDH), creatine kinase (CK), albumin, ferritin, leucocyte count, lymphocyte count, eosinophil count, hemoglobin, platelet count, erythrocyte sedimentation rate (ESR), fibrinogen, and L-dimer. 

Data analysis was conducted with STATA 16.1 program (StataCorp. 2019. Stata Statistical Software: Release 16. TX: StataCorp LP. USA). Differences in serological biomarker mean values between COVID-19 survivors with and without sensitization-associated symptomatology were assessed with a one-way ANCOVA, adjusted by gender and age. The level of significance was set at a priori 0.05. In addition, a correlation analysis with Pearson (r) coefficients was used to determine the association between CSI score and serological biomarkers. All statistically significant variables associated with CSI score were included into a generalized logistic multivariate regression model to identify those biomarkers contributing significantly to the variance of CSI score. The significance criterion of the F value for entry into the regression equation was set at *p* < 0.05. Changes in adjusted *R*^2^ were reported after each step of the regression model to determine the association of the additional variables.

## 3. Results

Details of the recruitment process and the demographics of the sample can be found elsewhere [9]. From 77 individuals initially evaluated, serological biomarkers data were obtained from 67 (87%), which were included in this analysis. No differences in clinical data between the included patient in the original paper and those included here were observed. Participants were assessed a mean of 6 (SD 0.8) months after hospitalization. Twenty-four (35.8%) patients reported sensitization-associated post-COVID-19 symptoms (CSI ≥ 40/100 points). Subjects reporting sensitization-associated symptoms had lower ferritin (*p* = 0.035) and hemoglobin (*p* = 0.015) levels than those not reporting sensitization-associated post-COVID-19 symptoms; however, the differences were small (Table 1). 

Significant but small negative associations of the CSI score with ferritin (r: −0.251, *p* = 0.04) and hemoglobin (r: −0.292, *p* = 0.017) levels was observed. No other significant association was found. The logistic multivariate regression model revealed that hemoglobin levels were the only laboratory biomarker associated with sensitization-associated symptoms by explaining the 13.7% of the variance of CSI score (r^2^ adj: 0.137, *B =* −0.394, t = −2.935, *p* = 0.005).

## 4. Discussion

This secondary analysis found lower ferritin and hemoglobin levels at the acute phase of infection in individuals with sensitization-associated post-COVID-19 symptoms at 6 months after hospital discharge. No other associations between the analyzed serological biomarkers at admission and sensitization-associated post-COVID-19 symptoms were seen. In general, higher levels of several of the analyzed serological biomarkers, e.g., D-dimer concentration or [20] blood glucose [21], were associated with severe COVID-19. In addition, higher ferritin levels were also found in severe–critical COVID-19 patients [22]. Accordingly, our results would suggest that severity of the infection in the acute phase is not associated with the development of sensitization-associated post-COVID-19 symptoms. It is hypothesized that SARS-CoV-2-associated cytokine and interleukin storms may lead to the sensitization of pain pathways [23,24], and trigger nociplastic pain responses by altering the balance between the neuromodulation systems of nociception [25]. Other hypotheses suggest that post-COVID-19 pain symptoms can be related to deficient immune regulatory mechanisms [26]. The current analysis did not observe different immune responses during the acute phase of the infection depending on the development of sensitization-associated symptoms; however, studies evaluating the long-term immune response in long-COVID-19 patients are needed. Indeed, it remains possible that a more pronounced and longer immune response in the subacute or chronic phase contributes to central nervous system changes and subsequent nociplastic changes. To date, few data are available on the association between the level of different cytokines and post-COVID-19 pain symptoms, because collection and preparation of blood samples for cytokine analysis is more rigorous than for standard serological biomarkers. 

Additionally, it should be considered that emotional and social factors surrounding the COVID-19 outbreak could play a relevant role in the development of post-COVID-19 pain. In fact, social alarm, somatization, post-traumatic stress disorder, and fear or uncertainty about prognosis have been associated with a promotion and perpetuation of musculoskeletal post-COVID-19 symptoms [27,28,29]. Nevertheless, the association between laboratory biomarkers and emotional and cognitive aspects would be expected to be small. 

Some limitations should be considered. Firstly, our data are only applicable to previously hospitalized COVID-19 survivors. Secondly, we did not include people with preexisting pain symptoms. Thirdly, several inflammatory biomarkers, e.g., cytokines or interleukins, were not analyzed, which may exhibit stronger associations with sensitization-associated post-COVID-19 symptoms. Finally, the exclusive use of the CSI for inferring sensitization is not recommended because it overlaps with emotional constructs, and because a self-reported tool cannot capture the complexity of central nervous system impairment [30]. In fact, our original study identified an association between anxiety levels and CSI, supporting the overlap with emotional distress [9].

## 5. Conclusions

In conclusion, although ferritin and hemoglobin levels were lower in patients with sensitization-associated post-COVID-19 symptoms, most investigated serological biomarkers at hospital admission were not associated with sensitization-associated post-COVID-19 symptoms in previously hospitalized COVID-19 survivors. 

## Figures and Tables

**Table 1:** Laboratory biomarkers of COVID-19 patients according to the presence or absence of sensitization-associated symptoms.

	CSI ≥ 40 Points (*n* = 24)	CSI < 40 Points (*n* = 43)	*p*
Glucose (mg/mL)	105.3 (21.9)	117.1 (45.0)	0.394
Creatinine (mg/dL)	0.9 (0.45)	0.9 (0.3)	0.855
Alanine transaminase (ALT, U/L)	24.5 (12.5)	25.6 (10.5)	0.599
Aspartate transaminase (AST, U/L)	22.6 (7.5)	22.7 (6.0)	0.967
Lactate dehydrogenase (LDH, U/L)	215.2 (41.8)	205.2 (42.1)	0.346
Creatine kinase (CK, mg/dL)	0.9 (0.35)	1.3 (1.75)	0.153
Albumin (g/dL)	4.5 (0.3)	4.5 (0.2)	0.626
Ferritin (ng/mL) *	83.9 (67.6)	158.25 (161.8)	0.024 *
Leucocytes (×10^9^/L)	7.05 (1.6)	7.25 (1.5)	0.491
Lymphocytes (×10^9^/L)	3.2 (0.6)	3.2 (0.8)	0.899
Eosinophils (×10^9^/L)	3.25 (3.4)	2.4 (1.95)	0.223
Hemoglobin (g/dL) *	13.55 (1.6)	14.4 (1.2)	0.047 *
Platelets (×10^9^/L)	252.5 (66.1)	244.7 (54.4)	0.588
(ESR, mm/h)	11.6 (10.7)	11.9 (13.1)	0.908
Fibrinogen (mg/dL)	418.1 (74.5)	408.2 (94.0)	0.608
L-dimer (ng/mL)	591.25 (470.9)	535.5 (621.5)	0.734

ESR—erythrocyte sedimentation rate; *n*—number; SD—standard deviation. * significant differences between subjects with and without sensitization-associated symptoms (*p* < 0.05).

## Data Availability

All data derived from this study are reported here.

## References

[1] Fernández-de-las-Peñas C., Navarro-Santana M., Plaza-Manzano G., Palacios-Ceña C., Arendt-Nielsen L. (2022). Time course prevalence of Post-COVID pain symptoms of musculoskeletal origin in patients who had survived to SARS-CoV-2 infection: A systematic review and meta-analysis. Pain.

[2] Fernández-de-las-Peñas C., de-la-Llave-Rincón A.I., Ortega-Santiago R., Ambite-Quesada S., Gómez-Mayordomo V., Cuadrado M.L., Arias-Navalón J.A., Hernández-Barrera V., Martín-Guerrero J.D., Pellicer-Valero O.J. (2021). Prevalence and risk factors of musculoskeletal pain symptoms as long-term post-COVID sequelae in hospitalized COVID-19 survivors: A multicenter study. Pain.

[3] Oguz-Akarsu E., Gullu G., Kilic E., Dinç Y., Ursavas A., Yilmaz E., Zarifoglu M., Karli N., Pandemic Study Team (2021). Insight into pain syndromes in acute phase of mild-to-moderate COVID-19: Frequency, clinical characteristics, and associated factors. Eur. J. Pain.

[4] Ursini F., Ciaffi J., Mancarella L., Lisi L., Brusi V., Cavallari C., D’Onghia M., Mari A., Borlandelli E., Faranda Cordella J. (2021). Fibromyalgia: A new facet of the post-COVID-19 syndrome spectrum? Results from a web-based survey. RMD Open.

[5] Nijs J., George S.Z., Clauw D.J., Fernández-de-las-Peñas C., Kosek E., Ickmans K., Fernández-Carnero J., Polli A., Kapreli E., Huysmans E. (2021). Central sensitisation in chronic pain conditions: Latest discoveries and their potential for precision medicine. Lancet Rheumatol..

[6] Kosek E., Clauw D., Nijs J., Baron R., Gilron I., Harris R.E., Mico J.A., Rice A.S.C., Sterling M. (2021). Chronic nociplastic pain affecting the musculoskeletal system: Clinical criteria and grading system. Pain.

[7] Fitzcharles M.A., Cohen S.P., Clauw D.J., Littlejohn G., Usui C., Häuser W. (2021). Nociplastic pain: Towards an understanding of prevalent pain conditions. Lancet.

[8] Goudman L., De Smedt A., Noppen M., Moens M. (2021). Is central sensitisation the missing link of persisting symptoms after COVID-19 infection?. J. Clin. Med..

[9] Fernández-de-las-Peñas C., Parás-Bravo P., Ferrer-Pargada D., Cancela-Cilleruelo I., Rodríguez-Jiménez J., Nijs J., Arendt-Nielsen L., Herrero-Montes M. (2022). Sensitization symptoms are associated with psychological and cognitive variables in COVID-19 survivors exhibiting post-COVID pain. Pain Pract..

[10] Bethea J.R., Fischer R. (2021). Role of peripheral immune cells for development and recovery of chronic pain. Front. Immunol..

[11] Verma V., Sheikh Z., Ahmed A.S. (2015). Nociception and role of immune system in pain. Acta Neurol. Belg..

[12] Samprathi M., Jayashree M. (2021). Biomarkers in COVID-19: An up-to-date review. Front. Pediatr..

[13] Mayer T.G., Neblett R., Cohen H., Howard K.J., Choi Y.H., Williams M.J., Perez Y., Gatchel R.J. (2012). The development and psychometric validation of the central sensitization inventory. Pain Pract..

[14] Scerbo T., Colasurdo J., Dunn S., Unger J., Nijs J., Cook C. (2018). Measurement properties of the central sensitization inventory: A systematic review. Pain Pract..

[15] Woby S.R., Roach N.K., Urmston M., Watson P.J. (2005). Psychometric properties of the TSK-11: A shortened version of the Tampa Scale for Kinesiophobia. Pain.

[16] García Campayo J., Rodero B., Alda M., Sobradiel N., Montero J., Moreno S. (2008). Validation of the Spanish version of the Pain Catastrophizing Scale in fibromyalgia. Med. Clin..

[17] Herrmann-Lingen C., Buss U., Snaith R.P. (2011). Hospital Anxiety and Depression Scale—Deutsche Version (HADS-D).

[18] Herdman M., Gudex C., Lloyd A., Janssen M., Kind P., Parkin D., Bonsel G., Badia X. (2011). Development and preliminary testing of the new five-level version of EQ-5D (EQ-5D-5L). Qual. Life Res..

[19] Neblett R., Cohen H., Choi Y., Hartzell M.M., Williams M., Mayer T.G., Gatchel R.J. (2013). The Central Sensitization Inventory (CSI): Establishing clinically significant values for identifying central sensitivity syndromes in an outpatient chronic pain sample. J. Pain.

[20] Du W.N., Zhang Y., Yu Y., Zhang R.M. (2021). D-dimer levels is associated with severe COVID-19 infections: A meta-analysis. Int. J. Clin. Pract..

[21] Chen J., Wu C., Wang X., Yu J., Sun Z. (2020). The impact of COVID-19 on blood glucose: A systematic review and meta-analysis. Front. Endocrinol..

[22] Kaushal K., Kaur H., Sarma P., Bhattacharyya A., Sharma D.J., Prajapat M., Pathak M., Kothari A., Kumar S., Rana S. (2022). Serum ferritin as a predictive biomarker in COVID-19. A systematic review, meta-analysis and meta-regression analysi. J. Crit. Care.

[23] Mulchandani R., Lyngdoh T., Kakkar A.K. (2021). Deciphering the COVID-19 cytokine storm: Systematic review and meta-analysis. Eur. J. Clin. Investig..

[24] Coomes E.A., Haghbayan H. (2020). Interleukin-6 in Covid-19: A systematic review and meta-analysis. Rev. Med. Virol..

[25] Cascella M., Del Gaudio A., Vittori A., Bimonte S., Del Prete P., Forte C.A., Cuomo A., De Blasio E. (2021). COVID-Pain: Acute and late-onset painful clinical manifestations in COVID-19: Molecular mechanisms and research perspectives. J. Pain Res..

[26] Ryabkova V.A., Churilov L.P., Shoenfeld Y. (2019). Neuroimmunology: What role for autoimmunity, neuroinflammation, and small fiber neuropathy in fibromyalgia, chronic fatigue syndrome, and adverse events after human papillomavirus vaccination?. Int. J. Mol. Sci..

[27] Chaturvedi S.K. (2020). Health anxiety, health-related life events, and somatization during COVID-19 pandemic can increase chronic pain. Pain.

[28] Meulders A., Vlaeyen J.W.S., Evers A.W.M., Köke A.J.A., Smeets R.J.E.M., Van Zundert J.H.M., Verbunt J.M.C.F., Van Ryckeghem D.M.L. (2021). Chronic primary pain in the COVID-19 pandemic: How uncertainty and stress impact on functioning and suffering. Pain.

[29] Karos K., McParland J.L., Bunzli S., Devan H., Hirsh A., Kapos F.P., Keogh E., Moore D., Tracy L.M., Ashton-James C.E. (2020). The social threats of COVID-19 for people with chronic pain. Pain.

[30] Nijs J., Huysmans E. (2021). Clinimetrics: The Central Sensitisation Inventory: A useful screening tool for clinicians, but not the gold standard. J. Physiother..

